# Editorial: Chronic Inflammation and Neurodegeneration in Retinal Disease

**DOI:** 10.3389/fphar.2021.784770

**Published:** 2021-10-18

**Authors:** Francesco Petrillo, Carlo Gesualdo, Chiara Bianca Maria Platania, Michele D’Amico, Maria Consiglia Trotta

**Affiliations:** ^1^ Department of Ophthalmology, University of Catania, Catania, Italy; ^2^ Eye Clinic, Multidisciplinary Department of Medical, Surgical and Dental Sciences, University of Campania “Luigi Vanvitelli”, Naples, Italy; ^3^ Department of Biomedical and Biotechnological Sciences, School of Medicine, University of Catania, Catania, Italy; ^4^ Department of Experimental Medicine, University of Campania “Luigi Vanvitelli”, Naples, Italy

**Keywords:** chronic inflammation, neurodegeneration, retinitis pigmenstosa, age-related macular degeneration, diabetic retinopathy, glaucoma

Retinal diseases represent one of the main causes of blindness all around the world, with tremendous social and economic impact. A deeper description of their pathophysiology is essential for improving their diagnostic and therapeutic management. Chronic inflammation and neurodegeneration have been recently recognized as important players in retinal damage. However, the exact mechanisms through which the function of retinal pigment epithelium (RPE) and neurons is impaired, leading to photoreceptors cell death, have still to be clarified. Therefore, the aim of the present Research Topic is to highlight the most recent discoveries in the field of retinal chronic inflammation and neurodegeneration, in order to collect the best evidence and the novel pharmacological approaches in the management of retinal diseases. Several articles published in this Research Topic are devoted to the main retinal pathologies, such as retinitis pigmentosa (RP), age-related macular degeneration (AMD), diabetic retinopathy (DR) and glaucoma.

It is well known that RP is the most common inherited retinal dystrophy, with no specific therapy against its progression. It is characterized by a progressive dysfunction of the photoreceptors, due retinal oxidative stress, glial and vascular changes which affects first the rods and then the cones. A great attention has been recently focused on the role of microglial activation in RP. Indeed, glial alterations were evident in a mouse model of RP (rd1 mouse) (Gimeno-Hernández et al.). These were reversed by the administration of thioredoxin (TRX), which also decreased the photoreceptors death, by activating glutathione (GSH) pathway (Gimeno-Hernández et al.). Accordingly, in human RP, microglia activation was present in retinal nuclear layer of regions populated by death committed rods (Micera et al.).

Concerning AMD, it is a complex multifactorial degenerative disease and the leading cause of blindness in individuals over the age of 55 in developed countries. During AMD pathogenesis, RPE cells lose their integrity, also by interacting with lymphocytes and macrophages trough integrins. To this regard, specific integrin antagonists were used as a strategy to interfere in the RPE-lymphocyte crosstalk (Baiula et al.). Integrin antagonists reduced ARPE-19 cell death and expression of IL-1β, then representing a novel opportunity to fight dry AMD (Baiula et al.). Another molecule considered as a novel therapeutic option in AMD is the antioxidant agent paraoxonase-1 (PON1), whose activity has been reported to be negatively correlated with malondialdehyde (MDA) levels in patients with AMD, by inhibiting lipid peroxidation (Micera et al.).

Among the constitutional risk factors favoring AMD, age is certainly the main one. However, gender has emerged as an important additional risk factor. To this regard, biological sex was shown to influence the expression of different age-related miRNAs (Hermenean et al.). Interestingly, miR-27a-3p, miR-27b-3p, miR-20a-5p and miR-20b-5p levels correlated with the thickness of specific retinal layers (Hermenean et al.). Concerning the genetic AMD risk factors, new insights on the polymorphisms of complement factor H (*CFH*) gene, the greatest genetic variations associated to AMD, have been reported (Stravalaci et al.). Specifically, the overexpression of long pentraxin 3 (PTX3) protein in RPE cells, cultured in inflammatory AMD-like conditions, along with the formation of a stable ternary complex between PTX3, factor H and its target C3b, was evidenced. This reduced *CFH* dysregulation in AMD (Stravalaci et al.).

Noteworthy, DR is the leading cause of blindness in working-age individuals in industrialized countries and is favored by a combination of microvascular damage, neuroinflammation and neurodegeneration processes. DR microvascular damage is triggered by hyperglycemia and retinal hypoxia. Interesting insights in epigenetic regulation of vascular endothelial growth factor A (VEGFA) and placental growth factor (PlGF), both increased during retinal hypoxic conditions, have been reported by Lazzara et al. Particularly, in human retinal endothelial cells (HRECs) challenged with chemical hypoxic stimuli (CoCl2), the authors showed that a focused set of HypoxamiRs, which are regulated by HIF-1α dependent or independent mechanisms, correlated not only with VEGFA levels but also with expression of TGFβ signaling pathway genes. These findings suggest the use of novel pharmacological/molecular approach, such as antagomiRs and agomir, to counteract microvascular damage induced by hypoxia (Lazzara et al.).

Retinal neurodegeneration is another key event in the progression of DR. Indeed, the death of retinal ganglion cells (RGCs) provoked by retinal insult could lead to irreversible blindness, since retina is characterized by limited repair capacity. Recently, plasma rich in growth factors (PRGF) was evaluated in ocular models for its capacity to promote neural survival (Ruzafa et al.). Particularly, the effect of the PRGF obtained from human, as well as from pig blood, was tested in organotypic cultures from adult pig retinas. PRGF induced microglial migration to the outer nuclear layers as a sign of inflammation. Due to the presence of several pro-inflammatory cytokines (Ruzafa et al.). Moreover, gliosis, proliferation and RGC survival were not affected by PGRF in organotypic cultures of adult porcine retinas. This effect was further investigated on primary cell cultures of RGCs and Müller cells alone or in co-cultures (Ruzafa et al.). Interestingly, the authors showed a selective protective effect of PGRF on Müller cells, since RGC survival was reduced in presence or absence of Müller cells (Ruzafa et al.).

Finally, glaucoma is the second leading cause of irreversible blindness in the world. It is a multifactorial neurodegenerative disease, characterized by the progressive death of RGCs and optic nerve head cupping. Particularly, the progressive damage to the optic nerve fibers is favored by an imbalance between the neuroinflammatory and neuroprotective mediators. Furthermore, glaucoma can be caused by the neurotoxic effects of glutamate, nitric oxide and oxidative stress. In this context, it has been demonstrated that the pro-inflammatory status induced in chronic rat model of glaucoma, can be reduced through stimulation of δ-opioid receptor activation by a selective ligand, SNC-121 (Husain et al.). Specifically, SNC-121 completely inhibited the increase in pro-inflammatory cytokines (Husain et al.). Further pre-clinical and clinical studies are necessary to fully understand the mechanisms underlying the onset of glaucoma. In this line, interesting anatomical changes have been highlighted in different mouse strains affected by glaucoma (Fiedorowicz et al.). Particularly, C57Bl/6J and DBA/2J mice showed different ocular dimensions, correlated to intraocular pressure (IOP) values (Fiedorowicz et al.).

Overall, the topic in retinal neurodegeneration and chronic inflammation highlights new preclinical findings in rodent models of ocular pathologies and pave the way to new biomarkers, pharmacological targets and therapeutic tools in retinal disease diagnosis and management.

